# Impact of Simulated Knee Injuries on the Patellofemoral and Tibiofemoral Kinematics Investigated with an Electromagnetic Tracking Approach: A Cadaver Study

**DOI:** 10.1155/2018/7189714

**Published:** 2018-04-23

**Authors:** Björn Rath, Malte Asseln, Marcel Betsch, Andreas Prescher, Markus Tingart, Jörg Eschweiler

**Affiliations:** ^1^Department of Orthopaedic Surgery, University Hospital RWTH Aachen, Aachen, Germany; ^2^Chair of Medical Engineering, Helmholtz-Institute Aachen, RWTH Aachen University, Aachen, Germany; ^3^Institute of Molecular and Cellular Anatomy, RWTH Aachen University, Aachen, Germany

## Abstract

**Purpose:**

The purpose of this study was to evaluate the approach of using an electromagnetic tracking (EMT) system for measuring the effects of stepwise, simulated knee injuries on patellofemoral (PF) and tibiofemoral (TF) kinematics.

**Methods:**

Three cadaver knees were placed in a motion rig. EMT sensors were mounted on the patella, the medial/lateral femoral epicondyles, the tibial condyle, and the tibial tuberosity (TT). After determining the motion of an intact knee, three injuries were simulated and the resulting bony motion was tracked.

**Results:**

Starting with the intact knee fully extended (0° flexion) and bending it to approximately 20°, the patella shifted slightly in the medial direction. Then, while bending the knee to the flexed position (90° flexion), the patella shifted progressively more laterally. After transecting the anterior cruciate ligament (ACL), the base of the medial menisci (MM) at the pars intermedia, and the medial collateral ligament (MCL), individual changes were observed. For example, the medial femoral epicondyle displayed a medial lift-off in all knees.

**Conclusion:**

We demonstrated that our EMT approach is an acceptable method to accurately measure PF joint motion. This method could also enable visualization and in-depth analysis of in vivo patellar function in total knee arthroplasty, if it is established for routine clinical use.

## 1. Introduction

The knee joint, which can be divided into the patellofemoral (PF) joint and tibiofemoral (TF) joint, is one of the most complex joints of the human body. It allows the tibia to move relative to the femur, while supporting body's weight, making it essential for activities of daily living.

A thorough understanding of knee kinematics is necessary to analyse initial joint derangement and progressive knee degeneration. The effectiveness of the PF joint depends on the bone and soft tissue balance as well as the anatomy of the trochlear groove (the most important predictor of patella maltracking) [1, 2]. Since a healthy knee has a valgus alignment, the tension of the quadriceps muscle produces a lateral patella movement [3]. An imbalance of the muscle tension can lead to PF pain syndrome and joint instability [3]. PF pain syndrome is very common and is widely believed to be caused by abnormal patella motion [4, 5]. Patients often present symptoms related to instability or maltracking because the PF joint is incongruent and the patella shows a large amount of movement during overall knee flexion and extension [5, 6]. Abnormal patellar tracking and motion, respectively, which may progress with the onset of osteoarthritis, alter the mechanical interaction between patella and femur [4]. Patellar maltracking implies that the patella is not following a normal, stable path within the trochlear groove [6]. Furthermore, an imbalance of bony and soft tissue structures (e.g., quadriceps muscle) can lead to PF pain syndrome and instability. Since there exists an association between PF pathology and excessive lateral tracking of the patella, assessing the overall lateral line of pull of the quadriceps muscle relative to the patella is a meaningful clinical measure. Such a measure is referred to as the quadriceps angle or *Q*-angle. PF pain syndrome is a very common problem and is widely believed to be caused by abnormal motion of the patella [4, 5]. The exact cause of and optimal treatment for PF pain syndrome are debatable [7].

The contact load on the trochlear groove also displays a very important aspect for the optimal function of the knee joint. PF disorders are often caused by changes of the PF and TF joint kinematics [4, 8–10]. An accurate analysis of PF joint kinematics in relation to TF joint kinematics, along with a definition of physiological and pathological joint kinematics, has not yet been fully achieved in experimental or clinical conditions [4, 10, 11]. An understanding of physiological and pathological patellar motion can also be useful in the diagnosis and treatment of PF joint disorders [4, 11, 12].

Little data is available regarding objective information about the effects of pathological factors which influence the patellar tracking/motion or have relationships with pathological symptoms [6, 12]. The reason for this is that many variables affect the measurements of patellar tracking, ranging from different reference points to the method of muscle loading [11]. The anatomical complexity of the PF joint makes it difficult to isolate the effect of a single surgical procedure, considering that medial stabilizers, trochlear dysplasia, patellar height, and other factors are fundamental to defining patellar tracking. Also, most patellar instability patients have additional abnormalities of varying degrees [2].

Tracking studies have provided quantitative data about the patellar kinematics during knee flexion and the joint's stability under given loading conditions [14]. PF kinematics have been the main focus of research in studies about PF instability and pain syndrome, and, furthermore, previous studies have shown that electromagnetic tracking (EMT) is a viable method for measuring joint kinematics [15–22]. In contrast to an optical tracking system, the EMT system does not require line of sight, and it uses much smaller and lighter tracking sensors [23, 24]. This approach, tracking the patella with an EMT, was published by Hefzy et al. [25], Sakai et al. [3, 26], Bull et al. [27], Amis et al. [6], and Cheung et al. [28]; while Hefzy, Sakai, and Cheung used the 3 SPACE tracker system (FASTRAK, Polhemus, Inc., Colchester, VT, USA), Bull and Amis used the “flock of birds” (FOB) system (Ascension Technology, Burlington, Vermont, USA).

Specific factors that may affect tracking, like sensor weight, sensor height, or sensor connection, were identified and should be taken into account when designing PF and TF tracking studies. For example, when measuring the joint motion by static methods, a series of joint angles and orientations must first be measured at different flexion angles, and then the motion of the joint between these measured points must be virtually reconstructed. However, this reconstruction has limitations [11, 29]. The passive occupancy of the trochlea by the stationary patella may be an unreliable guide to actual kinematics [11]. Due to this, the assessments, which are made by taking images at fixed increments of flexion with a static patella, are potentially misleading [11]. The effects will increase in vivo when quadriceps muscle action is important. Furthermore, valid information is only available for each of the static positions, and what happens in the virtual reconstruction between these positions depends on the algorithm used. This could lead to differences in kinematics, when comparing the results of the static and dynamic analysis in the same subject. In conclusion, it is difficult to understand the mechanics of normal patellar tracking as described in the available literature because of the irregularities in measurement techniques [11, 30].

In order to legitimise the use of an EMT system to measure the passive PF and TF joint kinematics in six degrees of freedom (DOF) ex vivo and with physiological and simulated knee injuries, three hypotheses were examined:

- First, an EMT system is capable of acquiring the PF and TF kinematics simultaneously.
- Second, it is possible to acquire the physiological PF and TF motion behaviour with a combination of an EMT system and a motion simulator.
- Third, the investigation of effects of gradually simulated injuries on PF and TF kinematic is possible.

## 2. Materials and Methods

A preliminary experiment using an EMT approach for kinematic tracking was carried out on three fresh-frozen cadaver knees (mean age: 81.3 years, range: 78 to 83 years, two right knees (knees 1 and 3), one left knee (knee 2)). X-rays of each specimen were taken to verify the absence of severe abnormalities. A manual test was performed to prove the absence of ligamentous dissociation. The skin, underlying fat, and muscles (other than the distal quadriceps) were removed in order to accurately position the tracking sensors.

**Figure 1:**
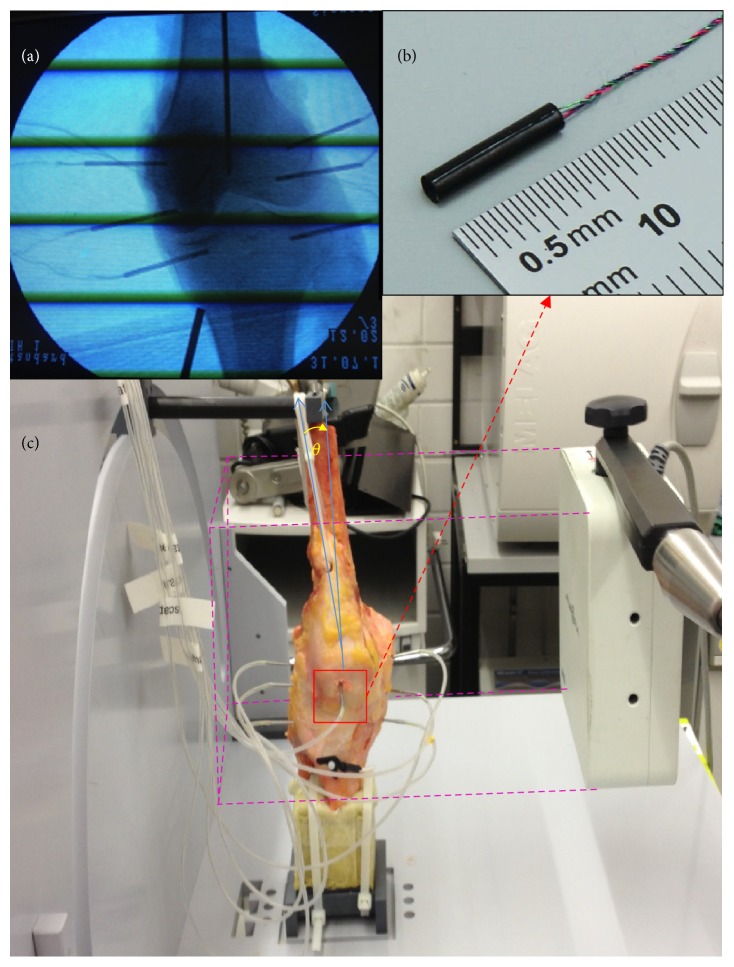
(a) The implanted sensors of the EMT system; (b) the dimension of one implanted sensor without the titanium tube covering; (c) an overview of the motion simulator and the test setup; *θ* = *Q*-angle.

**Figure 2:**
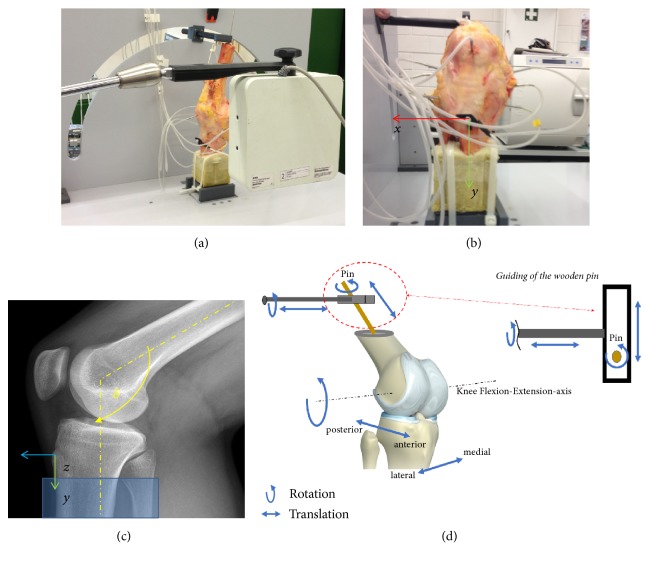
(a) The position of the cadaver specimen (right knee) in the simulator and the position of the EMT field generator; (b) the 90° flexed knee and the reference coil implanted in the TT; (c) the sagittal X-ray and orientation of the reference coordinate system; *δ* = knee flexion angle; (d) the degrees of freedom of the motion simulator.

The bony ex vivo kinematics were acquired in six DOF with an Aurora EMT system (Northern Digital Inc., [23]). The field generator (FG) (Figure 1(c), right side, and Figure 2(a)) emits a low-intensity, varying electromagnetic field and establishes the tracking volume (tracking volume: cubic volume of 500 × 500 [mm]) with a root mean square (RMS) of 0.48 mm for the position (translation) and 0.30° for the orientation (rotation) [23, 24, 31–34].

In order to avoid affecting the accuracy of the EMT system, the measuring rig was built entirely out of plastic (Figure 1(c)). EMT sensors (AURORA Mini 6 DOF (Northern Digital Inc.), see (Figure 1(b)), with a 1.8 mm diameter and a weight of 0.175 g including cable connection, [32]) were directly implanted press-fit via titanium tubes (with an outer diameter of 2.0 mm and an inner diameter of 1.8 mm) through small incisions into the medial and lateral femoral epicondyle. The sensors were placed 1 cm above the joint line. Additional sensors were put in both tibial condyles, and one sensor was put in the middle of the patella after drilling small holes (2.0 mm diameter). All sensor implantation was done under fluoroscopic vision. The sensor coils were embedded in titanium tubes for higher robustness against implied forces during sensor implantation and removal. The EMT approach allowed a continuous measurement of the angular and translational motion. The EMT device measured the position and orientation of a sensor in relation to a magnetic source with a measurement rate of 40 Hz.

The sensor, inserted in the tibial tuberosity (TT), was used to calculate the homogeneous coordinate transformations for all bones. The position of a rigid body {*A*} with respect to another coordinate system {*B*} can generally be represented by a 4 × 4 homogeneous transformation matrix **H** comprised of a rotation matrix and translation vector.

**Figure 3:**
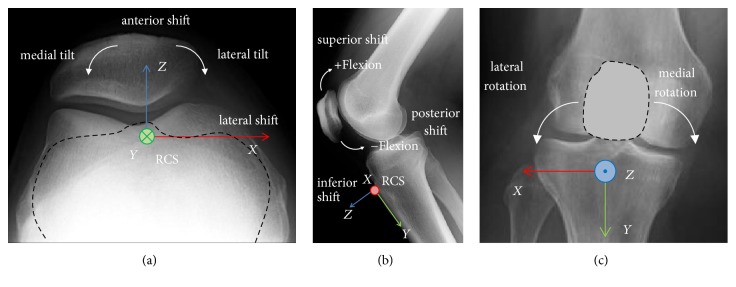
Patellar orientation and movement. The description of the motion patterns was taken from Bull et al. [13].

(1) HAB=RABpAorgB01,

where _*A*_^*B*^**R** is the 3 × 3 rotation matrix corresponding to the angles (*α*, *β*, *γ*) and ^*B*^**p**_*A*org_ is the origin of frame {*A*} in frame {*B*}. This approach was adopted to transfer the measurement data from the fixed EMT coordinate system, placed in the magnetic field generator itself, to the TT as a reference system. The medial/lateral direction was defined as the *x*-axis, positive direction towards the lateral side of the knee. The anterior/posterior direction was defined as the *z*-axis, positive in the anterior direction. Finally, the *y*-axis was defined as the line perpendicular to the *x*-axis and *z*-axis, positive direction towards the ground. For testing on a left knee, the positive *x*-axis runs towards the medial direction. The patella at 0° of knee flexion (fully extended) was defined as the reference configuration. The rotational behaviour of the patella was described by roll, pitch, and yaw. Roll is the rotation around the *x*-axis (patella flexion), pitch is the rotation around the *y*-axis (medial and lateral tilt), and yaw is the rotation around the *z*-axis (medial and lateral rotation). The orientation and movement (rotation and translation = shift), respectively, are shown and explained in Figure 3 in detail.

The tibia and fibula were cut distal to the tibia plateau, and both bones were moulded in polyurethane foam in an approximated physiological orientation. A framework around the femur allowed nylon pulleys to be mounted with cables running along the physiological path of the quadriceps muscle between the quadriceps tendon and the pulleys (see Figure 1(c)). A wooden pin was inserted into the femoral shaft to manually guide the femur during motion. This femur pin was connected to a guidance pin on the motion simulator. Free motion of the wooden femur pin was enabled to allow translation and rotation along and around the pin axis and translation along the simulator guidance pin (abduction/adduction) (Figure 2(d)). To avoid interferences, all potential error sources, for example, electric cabling, were placed out of the measurement field. The tendon of the quadriceps muscle was preloaded with 0.5 kg (approximately 5 N) to imitate the physiological basal tonus of the quadriceps muscle.

In order to verify that the EMT sensors and mounting hardware did not alter the physiological motions of the bones, the knee was moved manually. The motion rig (Figure 1(c)) allowed for a reproducible, passive joint motion in a flexion-extension/sagittal plane. The starting point of the recorded motion was full knee extension (0°) and the end point was 90° knee flexion. We know from our previous experiences that often patients have problems flexing the knee past 90° when certain complications arise, for example, a meniscus rupture. Overall motion was limited to 90° to ensure comparability. No external forces were applied to the test specimen. Each flexion-extension cycle from 0° to 90° took approximately 30 s. Each knee was flexed/extended three times with a recovery time of approximately 1 minute between each cycle [6].

The kinematic data was collected for the three knees with intact ligaments and then after sequential transecting of the anterior cruciate ligament (ACL), the base of the medial menisci (MM) at the pars intermedia, the dorsal horn, and the medial collateral ligament (MCL). The transecting of the ligaments and MM was performed by an experienced orthopaedic surgeon. ACL rupture is a common sports injury that can lead to possible degenerative joint changes [35, 36]. Additionally, we chose to simulate a meniscal tear because it is the most common knee injury, and partial meniscectomies are the most common orthopaedic surgical procedure [37]. An MCL tear also implies rotational trauma, where the foot is fixed on the ground and the femur rotates around the tibia (unhappy triad) [38].

After the kinematic data collection was completed, each knee was imaged via CT scan with an isotropic 0.7 voxel resolution. Based on the CT data, the correct position of each sensor relative to the bone was determined. Additionally, each specimen was anatomically dissected after the respective test series to verify that the sensors were placed correctly and that complete sectioning of the ligaments occurred.

## 3. Results

### 3.1. Patellofemoral Kinematics (Intact versus ACL Dissection)

**Figure 4:**
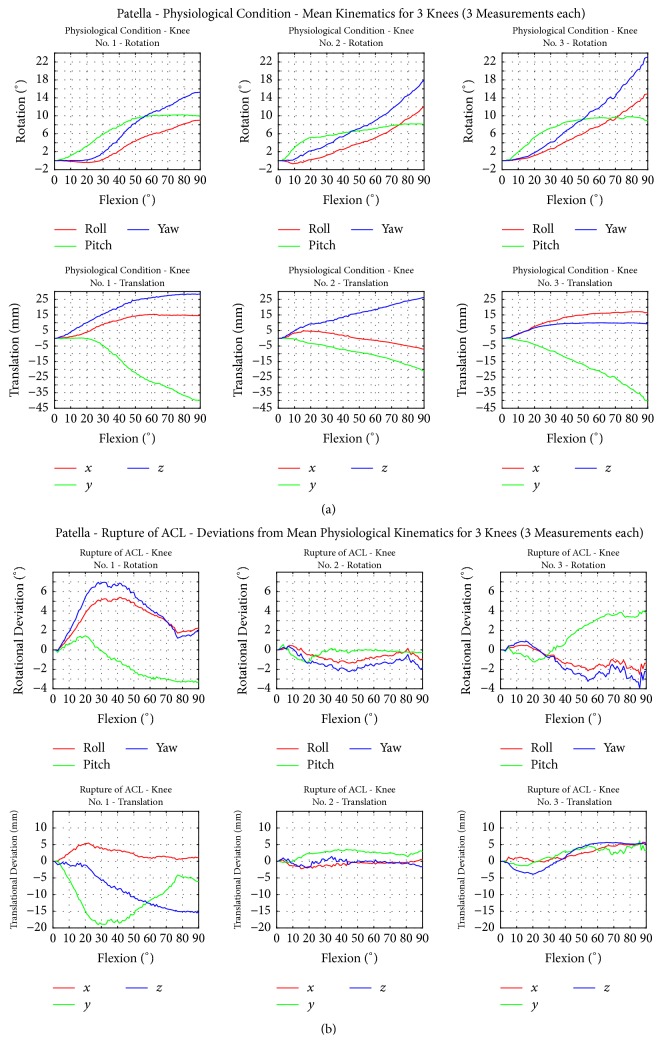
(a) Motion behaviour of the patella in the physiological case with intact ligaments. (b) Motion behaviour of the patella for simulated ACL rupture. The mean values of three repetitions for each knee are shown. Differences between the simulated ACL rupture and the physiological case are also shown.

Figure 4 demonstrates the patellar motion during flexion and extension of the TF joint from full extension to 90° flexion with intact ligaments (Figure 4(a)) and after cutting through the ACL (Figure 4(b)).

In two knees with intact ligaments, a lateral and superior shift of the patella was observed between 0° and 90° flexion. One knee showed a medial shift at 30° and a lateral shift at 60° and another at 90° flexion. All knees showed a superior shift at 30°, 60°, and 90° flexion with respect to the fixed tibia.

All patellae displayed an increasing medial rotation between 10° and 90° flexion. Between 10° and 90°, 20° and 90°, and 30° and 90°, superior (negative) flexion of the patella was observed.

After transection of the ACL, different patellar motion patterns were observed. The patella of knee 1 had a higher medial lift-off and superior (negative) flexion but less medial rotation when compared to the intact path. Knee 2 showed only small rotational changes, whereas knee 3 showed an inferior and lateral rotation at a flexion angle above 20° and an increase of the medial lift-off from 30° to 90°. Regarding the shifting, knee 1 displayed an increased superior and anterior shift (0°–90°) and an increased medial shift (10°–40°); knee 2 showed a small decrease of the superior shift and no changes in shifting in *x* and *z* directions; knee 3 showed a slight increase in medial, anterior, and inferior shift starting at 40° when compared to the intact situation.

### 3.2. Tibiofemoral Kinematics (Intact versus ACL Dissection)

**Figure 5:**
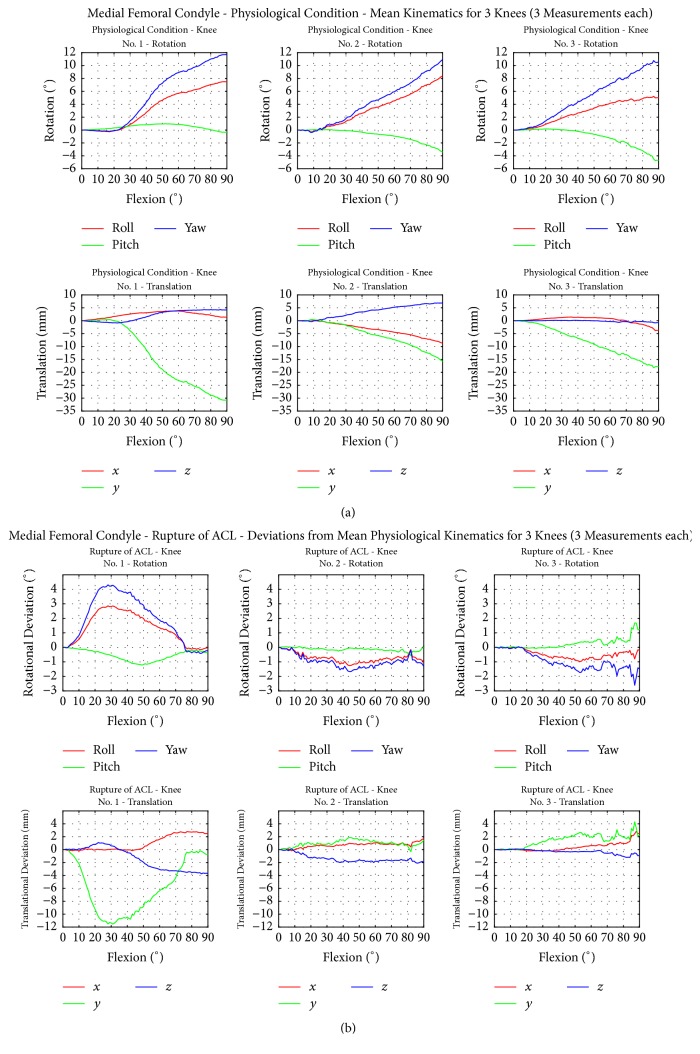
(a) Motion behaviour of the medial femoral condyle with intact ligaments. (b) Motion behaviour of the medial femoral condyle with simulated ACL rupture. Each graphic shows the mean physiological kinematics of knees 1 to 3 for three repetitions.

**Figure 6:**
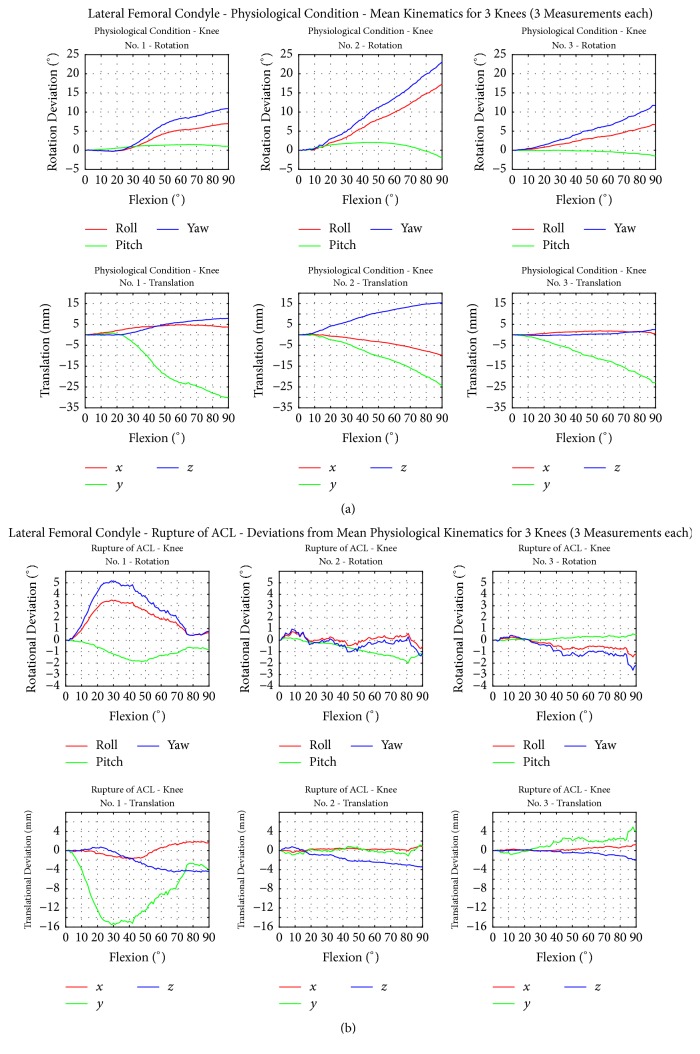
(a) Motion behaviour of the lateral femoral condyle with intact ligaments. (b) Motion behaviour of the lateral femoral condyle with simulated ACL rupture. Each graphic shows the mean physiological kinematics of knees 1 to 3 for three repetitions.

Figures 5 and 6 present the flexion-extension paths of motion of the medial and lateral femoral epicondyles of the TF joint under intact conditions and after transection of the ACL.

The medial femoral epicondyle displayed a medial lift-off in all knees starting at 20° flexion. The rotation around the *y*-axis (inner outer femoral rotation) differed in all knees. Knee 1 showed no rotation, whereas knees 2 and 3 showed anterior (outer) rotation.

In accordance with these results, the lateral femoral epicondyles displayed a lateral rotation around the *z*-axis and a positive rotation around the *x*-axis.

Knees 1 and 2 showed a slight lateral and anterior translation. Similar to the medial femoral epicondyle, all knees displayed a lateral lift-off and knees 1 and 2 additionally showed lateral and anterior translation during flexion. Knee 3 showed no changes in anterior-posterior or medial-lateral translation.

All knees with a transected ACL indicated small changes in femoral epicondyle motion during anterior-posterior rotation (inner outer femoral rotation) when compared to the intact condition. The medial and lateral condyle of knee 1 showed an increased lateral rotation in the abduction/adduction plane around the *z*-axis and an increased rotation around the *x*-axis (flexion-extension plane) compared to the intact knee. Knee 3 showed a slightly decreased rotation regarding the *x*-axis and *z*-axis beginning at 30° flexion.

Knee 1 displayed a higher anterior and lateral translation of the femoral condyles beginning at 50° flexion. The superior translation increased with a maximum at 30° flexion following a decrease up to 90°. Knee 3 indicated a decrease in the superior translation of both condyles after ACL transection and only minor changes regarding the *x*-axis and *z*-axis.

### 3.3. Patellofemoral and Tibiofemoral Kinematics (Intact versus ACL versus MM versus MCL Dissection)

**Figure 7:**
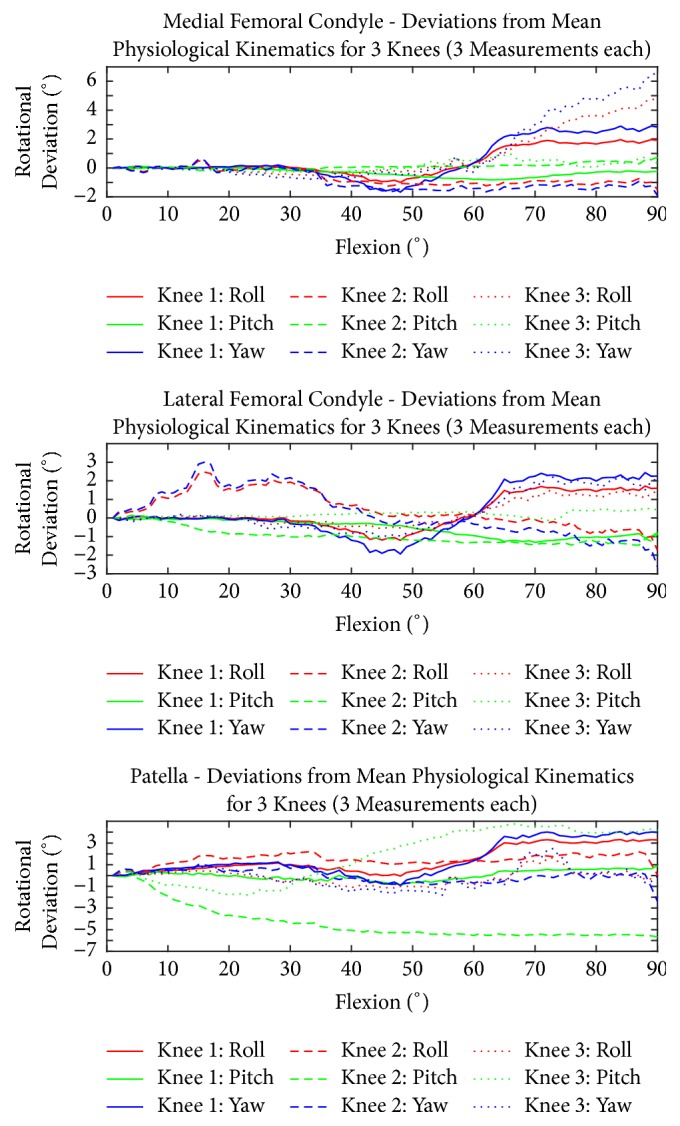
Rotational motion behaviour of the femoral condyles of the simulated, complex knee injury (ACL rupture, meniscus rupture, and MCL rupture) in comparison to patellar motion. The mean physiological kinematics for three repetitions and the differences compared to the physiological case are shown.

**Figure 8:**
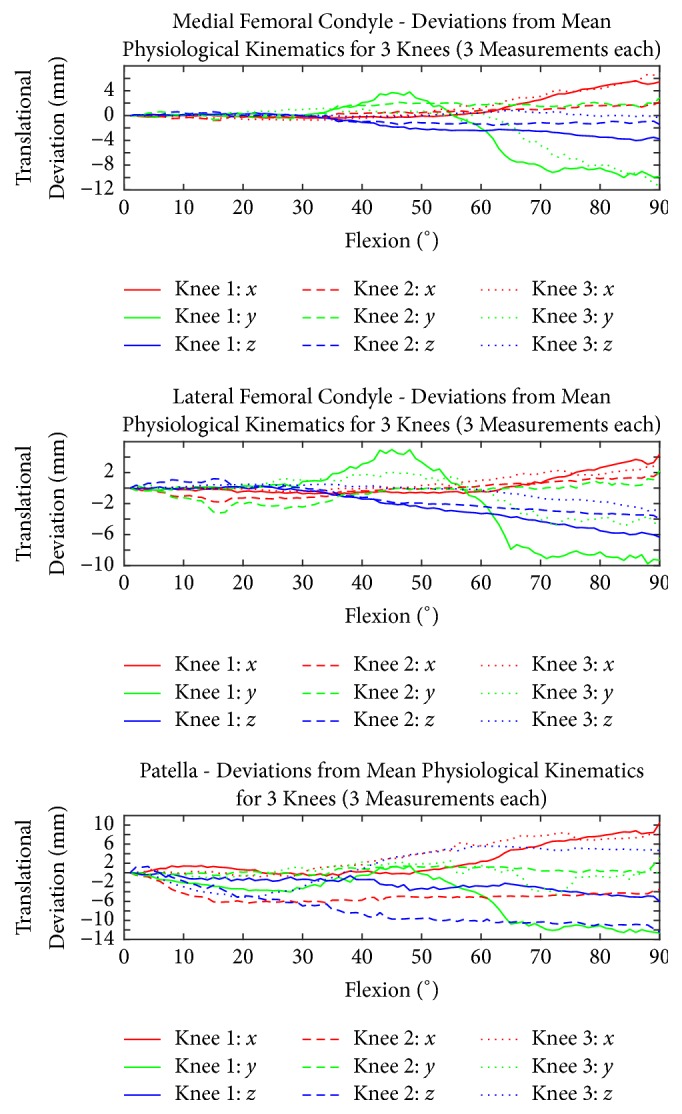
Translational motion behaviour of the femoral condyles of the simulated, complex knee injury (ACL rupture, meniscus rupture, and medial collateral ligament rupture) in comparison to the patellar motion. The mean physiological kinematics for three repetitions and the differences compared to the physiological case are shown.

Figures 7 and 8 show the PF and TF kinematics of the ACL, MM, and MCL.

When the ACL, MM, and MCL were dissected, each specimen showed individual changes in rotational and translational motion behaviour. The medial femoral condyles showed the highest increase in rotational and translational changes between 60° and 90° flexion. Knees 1 and 3 displayed the highest increase in medial lift-off and rotation. Knees 1 and 3 also showed an increase of the superior translation. The lateral femoral epicondyle of knees 1 and 3 displayed a decrease in lateral (outer) rotation and patellar flexion at an overall femoral flexion between 30° and 60° followed by an increase of both motion patterns between 60° and 90° flexion. Knee 2 displayed an increase in both rotational parameters at early flexion followed by a decrease between 60° and 90° overall flexion. The lateral femoral epicondyle of knee 2 showed a reduction in the superior shift between 30° and 60° following an increase starting at 60°. The lateral femoral epicondyle of knees 1 and 3 showed a higher lateral shift, while knee 2 displayed a lower lateral shift in accordance to the medial femoral epicondyles after ACL, MM, and MCL transection in reference to the intact joints.

The patellar motion displayed different patterns. The patella of knees 2 and 3 had a higher lateral lift-off. Knees 1 and 2 had a slight increase in superior (negative) flexion correlating with knee overall flexion. The anterior shift of the patella of knee 1 had almost no changes, whereas the patella of knee 2 had a decrease and knee 3 had an increase. All patellae displayed a higher medial shift during flexion when compared to the intact situation.

Regarding quantitative evaluation of the injury models (Table 1), for most of the arc of knee flexion, the patella followed a simple circular pattern around the trochlea. In the different figures (Figures 4(b), 5(b), 6(b), 7, and 8), it is possible to see that, in the injured cases, the deviation varied with the flexion angle. Compared to the physiological situation, in the extreme position of 90° flexion, the deviation increased in most of the cases.

## 4. Discussion

Disorders of the PF and TF joint are among the most challenging conditions encountered in orthopaedic practice. Accurately measuring PF and TF joint motion is important for understanding the effect of conservative and surgical treatment of knee pain syndrome [4]. In biomechanical investigations, the sequential positions of bones during joint motion are measured. Thus, absolute positions are not required, as a relative position of an individual bone is sufficient [27]. By choosing one reference system, it was possible to get the information of and relationship between both joints without transferring the patella motion to a different reference system. Also, the TF motion remains in its own reference system.

### 4.1. Kinematic Tracking Based on EMT

The advantage of using an EMT system was to achieve realistic measurements of the patellar tracking during knee flexion. We checked/proved the accuracy of the system by conducting our own tests (e.g., [22, 24]). During our investigation, we ensured that parameters that could influence the magnetic field were completely avoided. A minimally invasive, accurate measurement method needs to be established for routine clinical use.

### 4.2. Physiological PF and TF Kinematics

Many studies have reported PF and TF kinematics based on a sequence of measurements in static positions [8, 39, 40] (see also Table 1). Therefore, they may have failed to record transient effects [6]. Our study analysed PF and TF kinematics during a continuous, passive, and full range of knee movement (0°–90°).

One purpose of our study was to determine whether a general pattern for describing patellar tracking characteristics and TF kinematics could be established. For intact knees, in vitro, patellar tracking under the influence of tension of the quadriceps muscle followed consistent patterns. However, the range of normal tracking patterns may be large [11]. Our results are supported by literature [6, 27, 28].

Differences in the PF kinematics may be explained by the absence of active muscle forces in this ex vivo study. Additionally, there was a problem in comparing patellar tracking measurement results between different studies due to a difference in definition of the coordinate systems [6]. Furthermore, differences in rotational alignment and definition of the (functional) axes of the femur can lead to differences in the apparent medial-lateral shift, since the patella moves from anterior to posterior direction during femur flexion [6].

An analysis of the geometry of the distal femur showed the variability of the orientation of the trochlear groove in a group of normal knees [6, 41]. There exist anatomic differences between the specimen and knees; therefore, large numbers of subjects or cadaveric specimens should be used where possible. Knees with PF joint symptoms may have greater differences if there is some dysplasia [6].

### 4.3. Simulated Knee Injuries

Regarding the injuries, we found that the rotational and translational behaviour of the patella and femur increased. This is confirmed by the literature that states that these injuries cause a loss of stabilization qualities of the ligamentous apparatus [6].

It was discovered that the meniscus has an influence on the kinematics of the knee; however, this influence was not separately investigated as it was investigated in combination with the other simulated pathologies. If the meniscus is damaged, the kinematic motion of the knee changes as the motion of the patella increases. For example, Fan et al. found that an arthroscopic partial meniscectomy may result in early patellar maltracking [42]. This is a cause for patellofemoral pain and early degenerative arthrosis.

Our study makes it easier to understand the influence of different injuries on the motion behaviour of the medial and lateral condyle and, furthermore, on the patella. For the surgeon, it is important to know how the medial and lateral condyles interact with the corresponding parts of the tibia and influence the patellar motion. With this information, gathered from the implanted sensors, it is possible to get an individual impression of how pathological deviation of different injuries could be compensated or increase the deviation.

### 4.4. Limitations

Limitations of cadaveric studies are associated with the difficulty in simulating physiological muscle forces of active motion [7]. Clinical knowledge suggests that it is important to have experimental tracking data based on the physiological tensions and orientations of all the quadriceps muscle components. This could not be addressed in our passive investigation (preload of the quadriceps with 5 N only to simulate the basal muscle tonus). The influence of these factors on the measurement results was difficult to estimate. Further studies are strongly suggested to examine different active loading distributions, with loading as a function of flexion, in combination with knee injuries. This will help to understand the importance of soft tissue integrity on the PF and TF joint under more realistic situations.

Furthermore, the positioning of the specimens in the motion rig can cause slight differences of the detected trajectories. This is related to the fact that the distance between the receiver and transmitter must be kept within the optimal operational zone in order to minimize the positional error. This was taken into account for our test setup. Since only the knee joint was investigated, the influence of the adjoining hip and ankle joint (kinematic lower chain) on the knee's kinematic motion could not be addressed with our test setup.

We used the tibia as the central reference point; however, former studies have presented some problems with this approach. First, Katchburian et al. mentioned that this could be unsatisfactory because the main area of interest is the PF joint and the relative motion of its two components [11]. We used the tibia as our reference system because we also acquired the TF kinematics parallel to the PF kinematics.

Even the small sample size of three cadaver specimens in this study could have influenced the legitimacy of the presented motion behaviour. Evidently, interindividual differences will be reflected in the motion patterns.

## 5. Conclusion

The acquired motion changes from the intact to pathological situation might be useful to help clinicians evaluate the risk of continued instability and plan surgical approaches to improve PF and TF stability. In the future, this study should be repeated using an active motion simulator with additional specimens for statistical analyses and meaningful results.

In conclusion, we demonstrated that our EMT approach is useful for evaluating the intact situation and also pathologies of the knee. This method is a novel, minimally invasive, and lightweight approach to assess patellar kinematics and to achieve highly accurate measurements of patellar movements. This method could also enable visualization and in-depth analysis of in vivo patellar function in total knee arthroplasty if established for routine clinical use.

## Figures and Tables

**Table 1:** The maximum deviation magnitude for each trajectory in comparison to the physiological case. This is the most extreme point that was reached during the motion. For the mean calculation, we chose the absolute value.

	Knee 1			Knee 2			Knee 3			Mean			SD					
	*x*	*y*	*z*	*x*	*y*	*z*	*x*	*y*	*z*	*x*	*y*	*z*	*x*	*y*	*z*			
Patella	5.0	3.0	7.0	1.0	1.0	2.0	3.0	4.0	4.0	3.0	2.7	4.3	1.6	1.2	2.1	Rotation [ °]	Figure 4(b)	ACL rupture
	5.0	18.0	15.0	1.0	4.0	1.0	5.0	4.0	5.0	3.7	8.7	7.0	1.9	6.6	5.9	Translation [mm]	Figure 4(b)	ACL rupture
	3.0	1.0	4.0	2.0	6.0	2.0	1.0	5.0	2.0	2.0	4.0	2.7	0.8	2.2	0.9	Rotation [ °]	Figure 7	Complex injury
	10.0	12.0	6.0	6.0	4.0	12.0	8.0	4.0	6.0	8.0	6.7	8.0	1.6	3.8	2.8	Translation [mm]	Figure 8	Complex injury

Medial condyle	3.0	1.0	4.0	1.0	0.0	1.5	0.5	1.5	2.5	1.5	0.8	2.7	1.1	0.6	1.0	Rotation [ °]	Figure 5(b)	ACL rupture
	3.0	12.0	4.0	1.0	2.0	2.0	2.0	4.0	1.0	2.0	6.0	2.3	0.8	4.3	1.2	Translation [mm]	Figure 5(b)	ACL rupture
	2.0	0.5	3.0	1.0	0.0	1.0	5.0	0.0	6.0	2.7	0.2	3.3	1.7	0.2	2.1	Rotation [ °]	Figure 7	Complex injury
	6.0	10.0	4.0	2.0	2.0	1.0	6.0	12.0	0.0	4.7	8.0	1.7	1.9	4.3	1.7	Translation [mm]	Figure 8	Complex injury

Lateral condyle	3.5	2.0	5.0	0.5	2.0	1.0	1.5	0.5	2.5	1.8	1.5	2.8	1.2	0.7	1.6	Rotation [ °]	Figure 6(b)	ACL rupture
	2.0	16.0	2.0	1.0	1.0	4.0	1.0	5.0	2.0	1.3	7.3	2.7	0.5	6.3	0.9	Translation [mm]	Figure 6(b)	ACL rupture
	1.5	1.0	2.0	2.5	1.0	3.0	1.0	0.5	2.0	1.7	0.8	2.3	0.6	0.2	0.5	Rotation [ °]	Figure 7	Complex injury
	4.0	10.0	6.0	2.0	3.0	4.0	2.0	4.0	2.0	2.7	5.7	4.0	0.9	3.1	1.6	Translation [mm]	Figure 8	Complex injury
